# From gene detection to resistome ecology: targeted hybrid capture in AMR surveillance

**DOI:** 10.1128/msphere.00221-26

**Published:** 2026-06-15

**Authors:** Giulia Santarelli, Barbara Fiori, Cataldo Maria Mannavola, Roberto Rosato, Giordana Cafaro, Francesca Romana Monzo, Maurizio Sanguinetti, Flavio De Maio

**Affiliations:** 1 Department of Biotechnologies, Intensive Care and Perioperative Medicine, Catholic University of the Sacred Heart 96983, Rome, Italy; 2 Microbiota Analysis & Microbial WGS Research Core Facility, GSTeP, Fondazione Policlinico Universitario A. Gemelli IRCCS, L.go A. Gemelli 18654, Rome, Italy; 3 Microbiology Laboratory Unit; Department of Laboratory and Hematologic Sciences, Fondazione Policlinico Universitario A. Gemelli IRCCS, L.go A. Gemelli 18654, Rome, Italy; Kanazawa Daigaku, Kanazawa, Japan

**Keywords:** gut resistome, carbapenemase genes, targeted hybrid capture next-generation sequencing

## Abstract

Antimicrobial resistance (AMR) surveillance requires approaches capable of capturing the full resistome beyond culture-based and targeted molecular diagnostics. Here, we compared a commercial real-time PCR assay for carbapenemase detection with a targeted hybrid capture next-generation sequencing (xHYB) approach for comprehensive antimicrobial resistance gene (ARG) profiling in rectal swab samples. The xHYB panel showed high concordance with PCR for *bla_KPC_* and *bla_NDM_*, with overall agreement varying by gene target. Sequencing demonstrated higher analytical sensitivity, detecting additional ARGs not identified by PCR, particularly among *OXA*-like variants. Sequencing read counts were inversely correlated with PCR cycle threshold values, supporting a semi-quantitative relationship between methods, while no association was observed with total DNA concentration. Beyond single-gene detection, resistome analysis revealed significant compositional differences between carbapenemase-positive and -negative samples. Positive samples displayed a broader multidrug resistance profile, including β-lactamases, aminoglycoside-modifying enzymes, tetracycline, sulfonamide, macrolide, trimethoprim resistance genes, and multidrug efflux systems. Comparative analyses across resistance databases showed variability at gene and peptide-marker levels, whereas gene-family-level profiles were more consistent. Overall, targeted xHYB sequencing enables sensitive detection of clinically relevant ARGs and reveals structured resistome patterns associated with carbapenemase carriage, supporting its integration into AMR surveillance strategies. The xHYB panel showed high concordance with PCR for *bla_KPC_* and *bla_NDM_*, with overall agreement varying by gene target. Sequencing demonstrated higher analytical sensitivity, detecting additional ARGs not identified by PCR, particularly among *OXA*-like variants. Sequencing read counts were inversely correlated with PCR cycle threshold values, supporting a semi-quantitative relationship between methods, while no association was observed with total DNA concentration.

**IMPORTANCE:**

Antimicrobial resistance is a growing global health threat driven by the spread of resistance genes among bacteria, particularly in the human gut, which acts as a major reservoir. This study focuses on intestinal antimicrobial resistance genes from human rectal swabs, highlighting that the presence of carbapenemases was associated not only with single resistance genes but with a broader and more complex multidrug resistance profile. This indicates that conventional diagnostic methods may underestimate the true resistance potential of microbial communities. By providing a more comprehensive view of resistance gene diversity, this approach can improve surveillance and help identify high-risk resistance patterns earlier. These findings support the use of sequencing-based methods to complement existing diagnostics and strengthen monitoring of antimicrobial resistance in clinical and public health settings.

## INTRODUCTION

Antimicrobial resistance (AMR) represents one of the most pressing global health threats of the 21st century. In 2019 alone, bacterial AMR was associated with an estimated 4.95 million deaths worldwide, including 1.27 million deaths directly attributable to resistant infections (1). The rapid dissemination of antimicrobial resistance genes (ARGs) across clinical, environmental, and agricultural settings underscores the need for sensitive and scalable surveillance strategies capable of capturing the resistome beyond culture-based diagnostics (2). Unfortunately, understanding the burden of ARGs, the associated pathogens, and the leading pathogen-drug combinations contributing to their dissemination remains a crucial step for the prevention of the infections caused by multidrug-resistant (MDR) bacteria, as well as to manage surveillance control programs and ensuring access to essential antibiotics (3).

Despite the great efforts of the last years, there are important gaps in the data derived from many countries, especially low-income settings, emphasizing the need to expand microbiology laboratory capacity and data collection systems to improve our understanding of this topic.

Although we recognize the major leading pathogens for deaths associated with AMR in *Escherichia coli*, *Staphylococcus aureus*, *Klebsiella pneumoniae*, *Streptococcus pneumoniae*, *Acinetobacter baumannii*, *Pseudomonas aeruginosa*, the broad spectrum of ARGs associated with them makes it difficult to generate an ultimate identification panel (4).

Conventional shotgun metagenomic sequencing, although comprehensive, often lacks sufficient sensitivity for detecting low-abundance ARGs in complex environmental matrices, particularly in samples with low microbial biomass (5). In this context, rectal swabs provide a variable but generally consistent microbial load and directly reflect the gastrointestinal tract, which represents the main reservoir of MDR bacteria (6). Indeed, recent comparative studies have demonstrated that rectal swabs are a reliable proxy for fecal samples in microbiome analyses, showing no significant differences in alpha and beta diversity, preservation of inter-individual variability, and strong concordance in both taxonomic composition and inferred functional profiles. Additionally, metabolomic analyses have revealed an overall high correlation between rectal swabs and stool samples, further supporting their applicability for integrated microbiome studies (7). At the same time, they are readily available at the patient’s bedside and do not depend on specific timing, unlike stool samples (6). However, despite these advantages, rectal swabs remain strongly operator-dependent, and when not properly collected, they may not be fully representative, even though containing valuable levels of ARGs and bacterial load (6, 8). In clinical practice, inadequate sampling, often defined by the absence of visible fecal material, can lead to sample rejection and reduced screening efficiency, with a proportion of patients not being promptly re-screened (9). To address this issue, molecular markers have been proposed as internal controls for sampling adequacy. Among these, 16S rDNA represents a particularly robust candidate, as it reflects bacterial biomass and is less influenced by variability in host DNA content (9). On the other side, the sequencing depth remains a significant limitation for reliable ARGs detection, especially when searching for their association with metagenomic-assembled genomes, resulting in a time-consuming and expensive method (10).

To overcome these limitations, multiplex hybrid capture target enrichment (xHYB), implemented in the QIAseq xHYB AMR panel, has been developed to selectively enrich ARG targets prior to next-generation sequencing (NGS). This approach increases analytical sensitivity while maintaining broad resistome coverage. Multiple comparative studies have observed that hybrid capture significantly enhances ARGs detection compared to shotgun sequencing, especially for environmental specimens, by enabling the detection of clinically critical genes at low abundance levels (11). Its applications span hospital effluent surveillance, municipal wastewater monitoring, food industry environmental assessment, and urban AMR epidemiology (12).

In this study, we compare standard culture-based workflows and targeted molecular detection with xHYB for the detection of ARGs and evaluate the overall resistome in rectal swab specimens collected for the conventional routine AMRs surveillance.

## RESULTS

### Distribution of carbapenemase genes and isolated bacterial species in our cohort

A total of 120 samples were analyzed using AB Analitica (ABA) real-time PCR screening detection, including 74 positive (61.7%), 44 negative (36.7%), and 2 non-determined (ND, 1.6%) samples. Among the positive samples, carbapenemase gene group a + d (*bla_KPC_* and/or *bla_OXA-48-like_*) was detected in 50 cases (67.6%), with a mean cycle threshold (CT) value of 26.26 ± 5.00 (median 26.4; IQR 22.38–29.90); metallo-β-lactamase gene group b (*bla_VIM_*, *bla_NDM_*, and *bla_IMP_*) in 35 cases (47.3%), showing a higher mean CT value of 31.91 ± 8.30 (median 34.7; IQR 27.10–38.30), and *Acinetobacter OXA-like* genes (*AcOXA*) in 13 cases (17.6%) with a mean CT of 33.17 ± 4.65. Within this group, 43/50 isolates (86%) carried a + d genes, 15/35 (42.9%) carried b genes, and 7/13 (53.8%) harbored *AcOXA* genes.

The most frequently isolated species was *Klebsiella pneumoniae*, accounting for 47/74 positive samples (63.5%). Other identified species included *Acinetobacter baumannii* (2.7%), *Escherichia coli* (1.4%), and *Pseudomonas aeruginosa* (4.1%). Mixed infections were also observed, primarily involving *K. pneumoniae* in combination with other *Enterobacterales* or non-fermenting gram-negative bacteria. Negative samples (*n* = 44) showed no detection of carbapenemase genes (100%). ND samples (*n* = 2) were excluded from gene-specific analyses (Table 1).

**TABLE 1:** Distribution of positive, negative, and non-determined (ND) samples according to AB Analitica (ABA) carbapenemase gene detection and the associated isolated bacterial species^*a*^

Samples (no.)	Isolated species	No. (%)	a + d no. (%)	b no. (%)	AcOXA no. (%)
Positive (74)			50 (67.6)	35 (47.3)	13 (17.6)
	*A. baumannii*	2 (2.70)	–^*b*^	1 (2.86)	2 (15.4)
	*E. coli*	1 (1.35)	–	1 (2.86)	–
	*K. pneumoniae*	47 (63.5)	43 (86.0)	15 (42.9)	7 (53.8)
	*K. pneumoniae, A. baumannii*	1 (1.35)	–	1 (2.86)	1 (7.69)
	*K. pneumoniae, C. freundii*	1 (1.35)	1 (2.00)	–	–
	*K. pneumoniae, C. koseri*	1 (1.35)	1 (2.00)	1 (2.86)	1 (7.69)
	*K. pneumoniae, E. coli*	2 (2.70)	2 (4.00)	1 (2.86)	–
	*K. pneumoniae, P. aeruginosa*	1 (1.35)	1 (2.00)	1 (2.86)	–
	*P. aeruginosa*	3 (4.05)	–	3 (8.57)	–
	Negative	15 (20.3)	2 (4.00)	11 (31.4)	2 (15.4)
Negative (44)	Negative	44 (100%)	0	0	0
ND (2)	*P. aeruginosa*	1 (50%)	0	0	0

^*a*^ Genes “a + d” correspond to *bla_KPC_* and/or *bla_OXA-48-like_*, “b” to metallo-β-lactamase genes (*bla_VIM_*, *bla_NDM_*, and *bla_IMP_*), and “*AcOXA*” correspond to *Acinetobacter OXA-like* genes. Data are presented as absolute count and percentage. Frequencies reported in the first row are calculated over the total number of positive samples (*n* = 74). Percentages reported for each bacterial species within gene groups (a + d, b, *AcOXA*) are calculated using the number of samples positive for the corresponding gene group as the denominator (*n* = 50 for a + d, *n* = 35 for b, and *n* = 13 for *AcOXA*). Negative samples (*n* = 44) showed no carbapenemase gene detection. ND samples (*n* = 2) were excluded from further analysis.

^*b*^ "–" indicates that the corresponding data were not determined.

In the characterization panel, *bla_KPC_* was confirmed in 40 samples, with a mean CT of 24.34 ± 5.33 (median 23.9; IQR 21.18–27.38), while *bla_OXA-48-like_* genes were identified in 9 samples (mean CT 24.86 ± 4.65). Among metallo-β-lactamases, *bla_NDM_* was detected in 11 samples (mean CT 24.75 ± 7.00), and *bla*_VIM_ in 13 samples (mean CT 28.42 ± 6.67). *Acinetobacter* OXA-like genes were confirmed in three samples, with a mean CT of 25.80 ± 3.10 (Table 2).

**TABLE 2:** Screening and characterization tests for carbapenemase genes detection with corresponding cycle threshold (CT) values*^a^*

Panel	CT (mean ± SD; median [IQR])
Screening panel	
a + d, *n* = 50 (67.6%)	26.26 ± 5.00; 26.4 [22.38–29.90]
b, *n* = 35 (47.3%)	31.91 ± 8.30; 34.7 [27.10–38.30]
*AcOXA*, *n* = 13 (17.6%)	33.17 ± 4.65; 26.4 [33.20–36.60]
Characterization panel	
*bla_KPC_*, *n* = 40 (80.0%)	24.34 ± 5.33; 23.9 [21.18–27.38]
*bla_OXA-48 like_*, *n* = 9 (18.0%)	24.86 ± 4.65; 24.7 [21.30–29.20]
*bla_NDM_*, *n* = 11 (31.4%)	24.75 ± 7.00; 22.8 [19.70–27.63]
*bla_VIM_*, *n* = 13 (37.1%)	28.42 ± 6.67; 26.6 [22.10–34.70]
*OXA-like*, *n* = 3 (23.1%)	25.80 ± 3.10; 24.7 [23.40–29.30]

^*a*^ The screening panel detects gene groups a + d (*bla_KPC_* and/or bla_OXA-48-like_), b (*bla_NDM_*, *bla_VIM_*, and *bla_IMP_*), and *AcOXA* (*Acinetobacter OXA-like*), while the characterization panel identifies individual carbapenemase genes. Results are expressed as samples absolute count and percentages, with CT values reported as mean ± standard deviation (SD) and median with interquartile range (IQR).

Overall, the commercial panel showed a high prevalence of a + d genes, predominantly associated with *K. pneumoniae*, while characterization analysis confirmed *bla_KPC_* as the most frequently identified carbapenemase gene.

### Distribution of antibiotic resistance genes across databases by using xHYB panel

The evaluation of ARGs using the xHYB panel was performed on previously described benchmark rectal swab samples. For each detected ARG, information regarding peptide markers, gene annotations, Antibiotic Resistance Ontology (ARO) genes, and ARO phenotypes was retrieved for downstream analysis. Additionally, genes were aggregated to generate a cumulative variable referred to as gene family.

**Fig 1:**
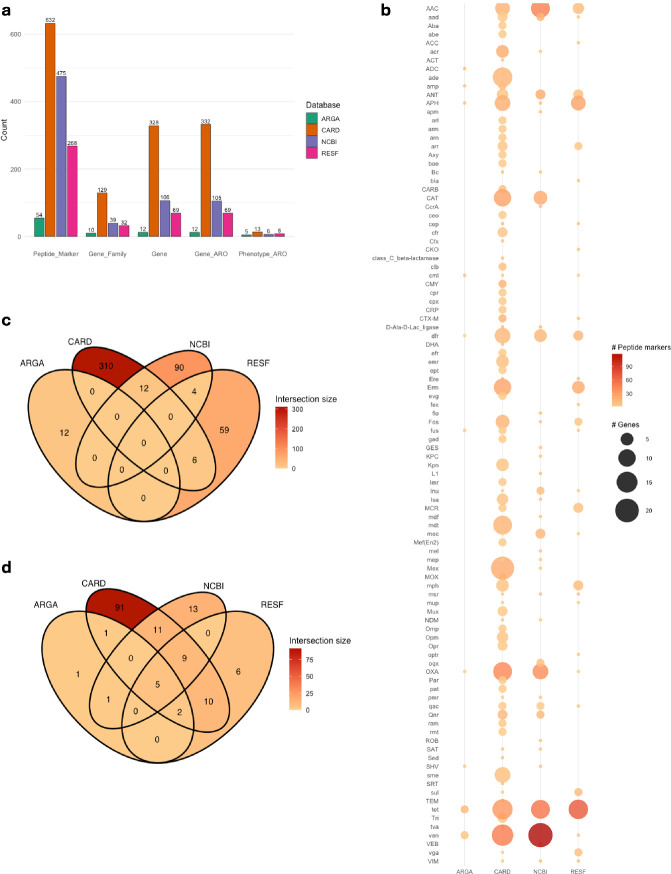
Performance of hybrid capture sequencing. (**a**) Distinct antimicrobial resistance entity counts across ARG databases obtained by hybrid capture (xHYB) analysis. For each database (ARGA, CARD, NCBI, and RESF), the number of individual peptide markers, ARGs, ARG families (aggregated ARGs), ARO gene annotations, and ARO phenotype annotations were computed. These values were summarized to highlight differences in entity coverage across databases by using a grouped bar plot. (**b**) Distribution of the ARGs across databases. Bubble plot represents the distribution of the ARG families (y-axis) across databases (x-axis). Bubble size represents the number of distinct genes per gene family, while color intensity indicates the number of associated peptide markers, enabling simultaneous visualization of gene-level coverage and peptide marker annotation depth. Full aggregated gene families table is provided as Data S3. To test whether databases differed in their distributions of gene coverage and peptide marker annotation depth, non-parametric comparisons were performed across databases using the Kruskal-Wallis test with Benjamini-Hochberg (BH) correction for each of the following metrics: genes, peptide markers, and peptide markers per gene. (**c**) Overlap of ARGs across databases. Venn diagram shows the overlap of distinct resistance genes among ARGA, CARD, NCBI, and RESF. Numbers indicate the count of genes in each intersection. (**d**) Overlap of resistance gene families across databases. Venn diagram shows the overlap of ARG families among ARGA, CARD, NCBI, and RESF. Numbers indicate the count of gene families in each intersection. Compared with the gene-level analysis, a larger shared core is observed, indicating a slight convergence at the ARG family level despite the substantial divergence in gene-level annotation across databases.

Marked differences in annotation coverage were observed among the databases used for ARG identification: Comprehensive Antibiotic Resistance Database (CARD), National Center for Biotechnology Information (NCBI), ResFinder database (RESF), and ARG analyzer (ARGA) (Fig. 1a). CARD exhibited the highest annotation depth, identifying 632 distinct peptide markers, 328 genes, 129 gene families, and 332 Gene ARO annotations. NCBI followed with 475 peptide markers, 106 genes, 39 gene families, and 105 Gene AROs. RESF and ARGA showed more limited detection capacity, with ARGA presenting the lowest counts across all categories. Phenotype ARO annotations were limited in all databases (range: 5–13). These results highlight substantial heterogeneity in annotation extensiveness, with CARD and NCBI providing considerably richer content compared to RESF and ARGA. Across all databases, most genes were supported by a single peptide marker. This trend was particularly pronounced in CARD, where a large proportion of genes were associated with only one marker, with progressively fewer genes supported by multiple markers. NCBI and RESF displayed similar, though less skewed, distributions, while ARGA showed a narrower range of marker support (Fig. S1a; Data S1). A chi-square test confirmed significant differences in peptide marker support distributions across databases (χ² test*, P* < 0.001), with a moderate effect size (Cramér’s *V* = 0.35). At the gene-family level, peptide marker distributions were broader than at the gene level (Cramér’s *V* = 0.43; χ² test*, P* < 0.001) (Fig. 1a).

When examining gene families, CARD and NCBI consistently showed broader coverage, characterized by larger numbers of genes per family and a higher density of associated peptide markers. In contrast, RESF and ARGA displayed fewer gene families and smaller gene counts, rather than substantially lower peptide marker abundance (Fig. 1b). Although the total number of genes per gene family did not differ significantly among databases (χ² *=* 3.48*,* df *=* 3*, P =* 0.323), significant differences were observed for: number of peptide markers (χ² *=* 25.89*,* df *=* 3*, P =* 1.00 *×* 10⁻⁵) and peptide marker-to-gene ratio (χ² *=* 59.49*,* df *=* 3*, P =* 7.55 *×* 10⁻¹³). These findings indicate that inter-database variability is primarily driven by differences in peptide-marker annotation depth rather than by gene assignment *per se*.

Spearman correlation analysis confirmed a positive association between gene number and peptide marker abundance within databases. Strong correlations were observed for: CARD (*ρ =* 0.71*, P =* 1.85 *×* 10⁻²¹), NCBI (*ρ =* 0.75*, P =* 2.93 *×* 10⁻⁸), and RESF (*ρ =* 0.72*, P =* 2.76 *×* 10⁻⁶). In contrast, ARGA showed a weaker and non-significant correlation (*ρ =* 0.35*, P =* 0.32), reflecting its comparatively limited annotation scope (Fig. 1b). At the gene level, correlations between peptide-marker support and total read counts were weaker overall (NCBI: *ρ =* 0.35*, P =* 0.0005, CARD: *ρ =* 0.02*, P =* 0.75, RESF: *ρ =* 0.56*, P =* 1.87 *×* 10⁻^6^, ARGA: *ρ =* 0.58*, P =* 0.064), with substantial dispersion of points and database-specific trends (Fig. S1b). Patterns observed for RPKM mirrored those for raw read counts (Fig. S1c). These results indicate that, while genes supported by more peptide markers tend to accumulate higher read counts, this relationship is not strictly proportional and differs across databases.

Comparison of shared ARGs across databases revealed limited overlap at the gene level (Fig. 1c). A substantial proportion of genes were database specific. While CARD contributed the highest number of unique genes, NCBI also showed a considerable number of unique entries. Finally, RESF and ARGA displayed smaller unique gene sets and minimal overlap with other databases. Only a small core of genes was shared across all four databases, indicating strong database dependency at the gene level. Pairwise overlap was most prominent between CARD and NCBI, whereas overlaps involving ARGA were consistently limited (Data S2).

In contrast, overlap increased when analyses were conducted at the gene-family level (Fig. 1d). Major resistance gene families, including APH, dfr, OXA, tet, and van, were shared across multiple databases, with a sizeable core present in all four resources. Although CARD contributed the largest number of families, NCBI, RESF, and ARGA also shared a substantial fraction despite gene-level discrepancies (Data S3).

**Fig 2:**
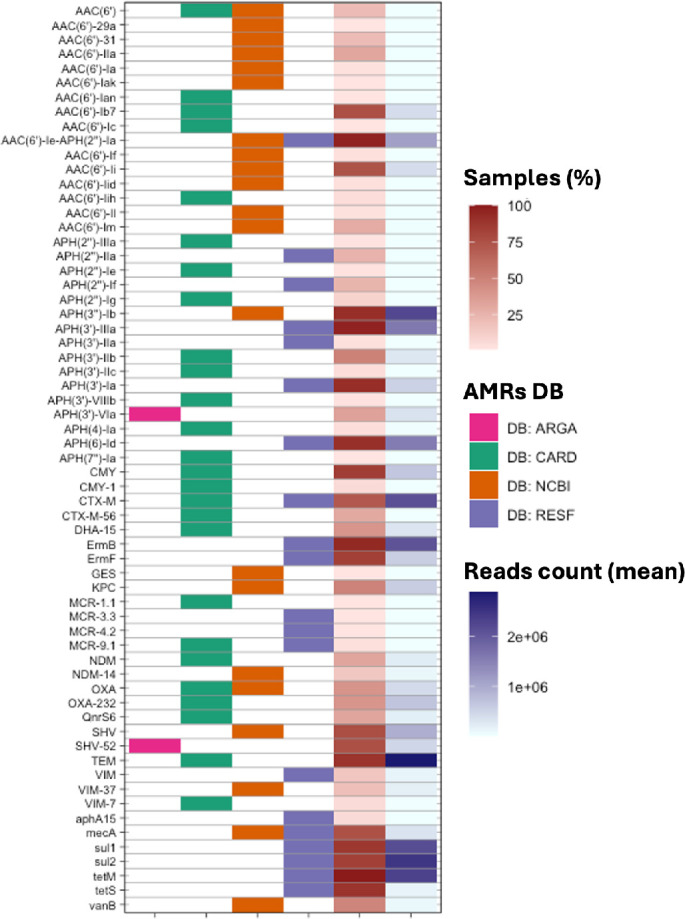
Schematic representation of the clinically relevant genes detected by hybrid capture (xHYB) panel. *bla_CTX-M_, bla_TEM_, bla_VIM_, bla_CMY_, bla_SHV_, GES, bla_OXA-48_, mcr, qnrS, aac(6′), aph, ermB/F, tetM/S, sul1/2, mecA,* and *vanB* have been highlighted. For each gene has been showed antimicrobial resistance databases (ARGA, CARD, NCBI, and RESF), reads count average, and percentage of positive samples.

A subset of clinically relevant ARGs (*bla_CTX-M_*, *bla_TEM_*, *bla_VIM_*, *bla_CMY_*, *bla_SHV_*, *GES*, *bla_OXA-48_*_-like_, *mcr*, *qnrS*, *aac(6′), aph*, *ermB/F*, *tetM/S*, *sul1/2*, *mecA*, and *vanB*), defined according to references 13, 14, was extracted from Data S1. For each gene, the following metrics were computed: (i) number and percentage of samples in which the gene was detected; (ii) mean read count across samples; and (iii) database provenance (ARGA, CARD, NCBI, and RESF) (Fig. 2; Data S3).

Several clinically relevant ARGs were supported by annotations from multiple databases, whereas others were uniquely represented in a single source. CARD and NCBI contributed the majority of annotations for clinically important genes, while RESF and ARGA provided more limited but complementary coverage. These findings reflect differences in database focus and curation strategies.

### Comparison xHYB panel versus AB Analitica commercial assay for detection of carbapenemase genes

**Fig 3:**
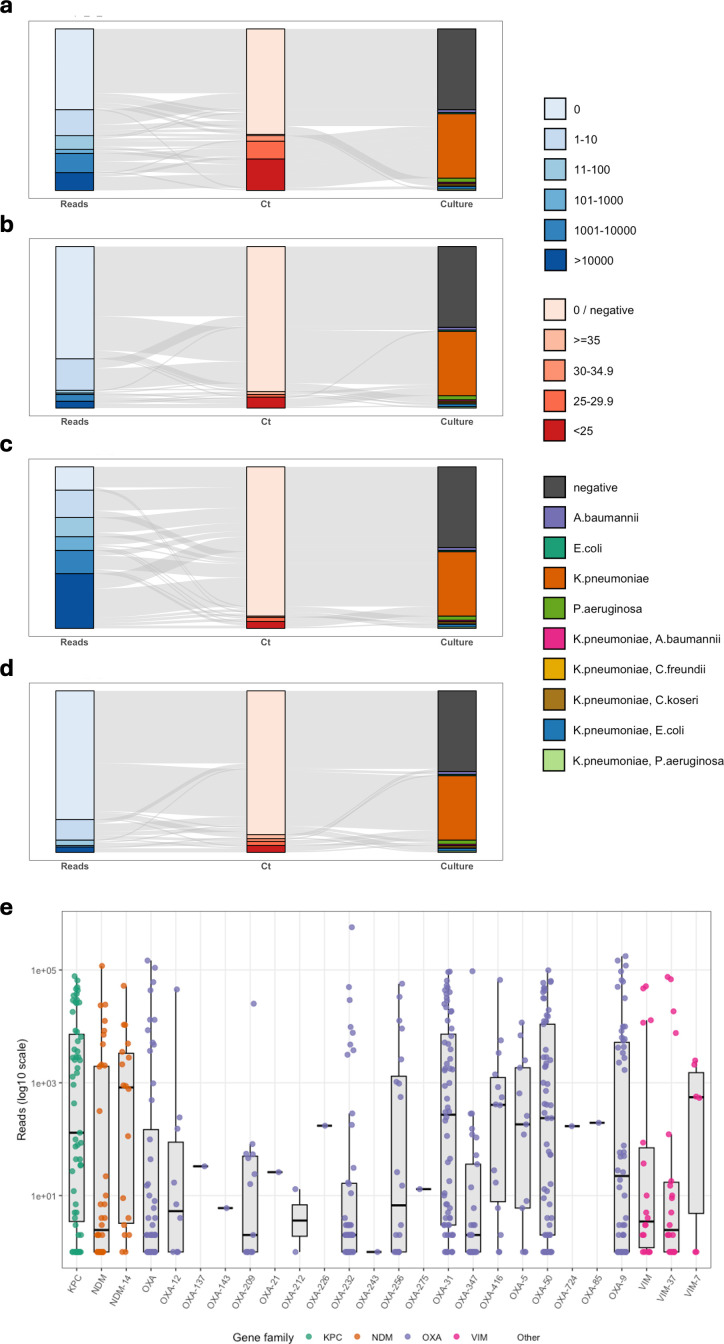
Distribution of resistance gene families across sequencing, PCR detection, and culture. For each sample, total read counts were calculated at the resistance-family level. Alluvial plots illustrating the relationship between sequencing read counts, PCR cycle threshold (Ct) values, and microbiological culture results for four carbapenemase gene families: *blaKPC* (**a**), *blaNDM* (**b**), *OXA* (**c**), and *blaVIM* (**d**). Sequencing read counts were grouped into abundance classes (0, 1–10, 11–100, 101–1,000, 1,001–10,000, and > 10,000). Ct values were categorized into five intervals (0, ≥35, 30–34.9, 25–29.9, and <25). Gray flows connect read-count classes to Ct categories and subsequently to culture results. Colored stratifications represent the individual classes for each analytical layer. In panel c, boxplots with overlaid points show the distribution of read counts for each gene subtype, ordered by family, across all samples (**e**). The y-axis is displayed on a logarithmic scale to account for the wide dynamic range of sequencing signals. Only detections with read counts greater than zero are shown.

The relationship between sequencing-derived read counts, PCR cycle threshold (Ct) values, and microbiological culture outcomes for the four major carbapenemase gene families (*bla_KPC_*, *bla_NDM_*, *bla_OXA_*, and *bla_VIM_*) was evaluated. Out of 120 samples, 118 were evaluable by both methods and included in the analysis, while two samples were excluded due to ND results (Table 1). Alluvial plots for each gene were generated (Fig. 3). Across all gene families, a clear relationship was observed between sequencing read abundance and Ct. Samples with higher read counts were generally associated with lower Ct values, indicating concordance between sequencing-based detection and molecular amplification signals. Particularly, for *bla_KPC_*, several samples showed moderate to high read counts and correspondingly low Ct values, with most culture-positive cases associated with *K. pneumoniae*, consistent with the known epidemiology of *bla_KPC_*-producing *Enterobacterales* (Fig. 3a). For *bla_NDM_*, overall read counts were lower compared with *bla_KPC_*, although a subset of samples exhibited high sequencing abundance together with low Ct values and positive cultures (Fig. 3b). The *bla_OXA_* family showed the widest distribution of read counts and the greatest subtype diversity (Fig. 3c). In several cases, relatively high sequencing signals were associated with negative or high Ct PCR values. This pattern reflects the presence of multiple *bla_OXA_* variants detected by sequencing, including subtypes not specifically targeted by the PCR assay. Finally, for *bla_VIM_*, fewer samples were positive, but those with detectable reads often showed concordant Ct values and culture-positive results (Fig. 3d). Low read-count observations were predominantly distributed in the 1–10 read range, supporting the application of a ≥10 read threshold to distinguish likely true signals from background sequencing noise.

The performance of xHYB panel in detecting carbapenemase genes was evaluated by comparison with the ABA test, which was used as the reference method. For each gene (*bla_KPC_*, *bla_OXA-48_*, *bla_NDM-1_*, and *bla_VIM_*), the results obtained by NGS were compared with those obtained by ABA and summarized using confusion matrices (Fig. S2a through d). To quantify the level of agreement between the two methods, standard diagnostic performance metrics were calculated, including positive percent agreement (PPA), negative percent agreement (NPA), positive predictive value (PPV), negative predictive value (NPV), overall accuracy, and Cohen’s kappa coefficient. For *bla_KPC_*, NGS showed a high level of concordance with ABA (Fig. S2a). Among the 118 isolates analyzed, 38 true positives and 75 true negatives were observed, with only two false positives and three false negatives. This corresponded to a PPA of 0.92 and a NPA of 0.97. The positive predictive PPV was 0.95, while the NPV reached 0.96. Overall accuracy was 0.96, and the Cohen’s kappa coefficient was 0.90, indicating almost perfect agreement between NGS and ABA for the detection of *bla_KPC_*. For *bla_NDM-1_*, NGS demonstrated very good agreement with the reference method (Fig. S2b). A total of 11 true positives and 104 true negatives were identified, with two false positives and one false negative. The resulting PPA was 0.92 and the NPA was 0.98. The PPV was 0.85, while the NPV was 0.99. The overall accuracy reached 0.97, and the kappa coefficient was 0.87, indicating strong agreement between NGS and ABA for *bla_NDM-1_* detection. For *bla_OXA-48_*, the agreement between the two methods was lower (Fig. S2c). NGS detected 9 true positives and 88 true negatives, but also produced 21 false positives, while no false negatives were observed. Consequently, the PPA was 1.00, indicating that all ABA-positive isolates were detected by NGS. However, the NPA was 0.80, reflecting the relatively high number of NGS positives not confirmed by ABA. The PPV was therefore low (0.30), whereas the NPV reached 1.00. The overall accuracy was 0.82, and the kappa coefficient was 0.390, indicating only fair agreement between NGS and ABA for *bla_OXA-48_* detection. For *bla_VIM_*, the level of agreement was moderate (Fig. S2d). NGS identified 7 true positives and 103 true negatives, with two false positives and six false negatives. The PPA was 0.54, indicating that approximately half of the ABA-positive isolates were detected by NGS. In contrast, the NPA was high (0.98), demonstrating strong agreement for negative samples. The PPV was 0.78, and the NPV was 0.94. Overall accuracy was 0.93, with a kappa coefficient of 0.600, corresponding to moderate agreement between NGS and ABA. Overall, the concordance between NGS and the reference ABA method varied depending on the carbapenemase gene analyzed. Agreement was excellent for *bla_KPC_* and *bla_NDM-1_*, moderate for *bla_VIM_*, and considerably lower for *bla_OXA-48_*, primarily due to a substantial number of NGS-positive results not confirmed by ABA. DNA concentration was then investigated according to the PCR results, revealing a substantial variability across samples, with overlapping distributions between ABA-negative and ABA-positive groups (Fig. S3a). This finding suggests that DNA yield did not impact the detection of carbapenemase resistance genes. Additionally, correlation analysis was performed to evaluate the relationship between PCR Ct values and NGS read counts for each carbapenemase gene (Fig. S3b). For the *bla_KPC_* gene, a statistically significant moderate negative correlation was observed (*ρ =* 0.468, *n* = 41, *P =* 0.002), suggesting concordance between PCR detection and NGS-based identification. In contrast, the correlation between Ct values and sequencing reads for the *bla_NDM_* gene was weak and not statistically significant (*ρ =* 0.154, *n* = 12, *P =* 0.633). A similar pattern was observed for *bla_OXA-48-like_* genes (*ρ =* 0.150, *n* = 9, *P =* 0.700). For the *bla_VIM_* gene, a slight negative but not significant correlation was observed (*ρ =* 0.314, *n* = 13, *P =* 0.295). These results suggest that variation in sequencing read counts is not primarily driven by differences in total DNA concentration within the analyzed samples. Taken together, these findings highlighted that even though NGS read counts exhibited highly skewed and heterogeneous distributions, the identified threshold allowed to discriminate carbapenemase-positive samples.

### Resistome structure differs between carbapenemase-positive and -negative samples

**Fig 4:**
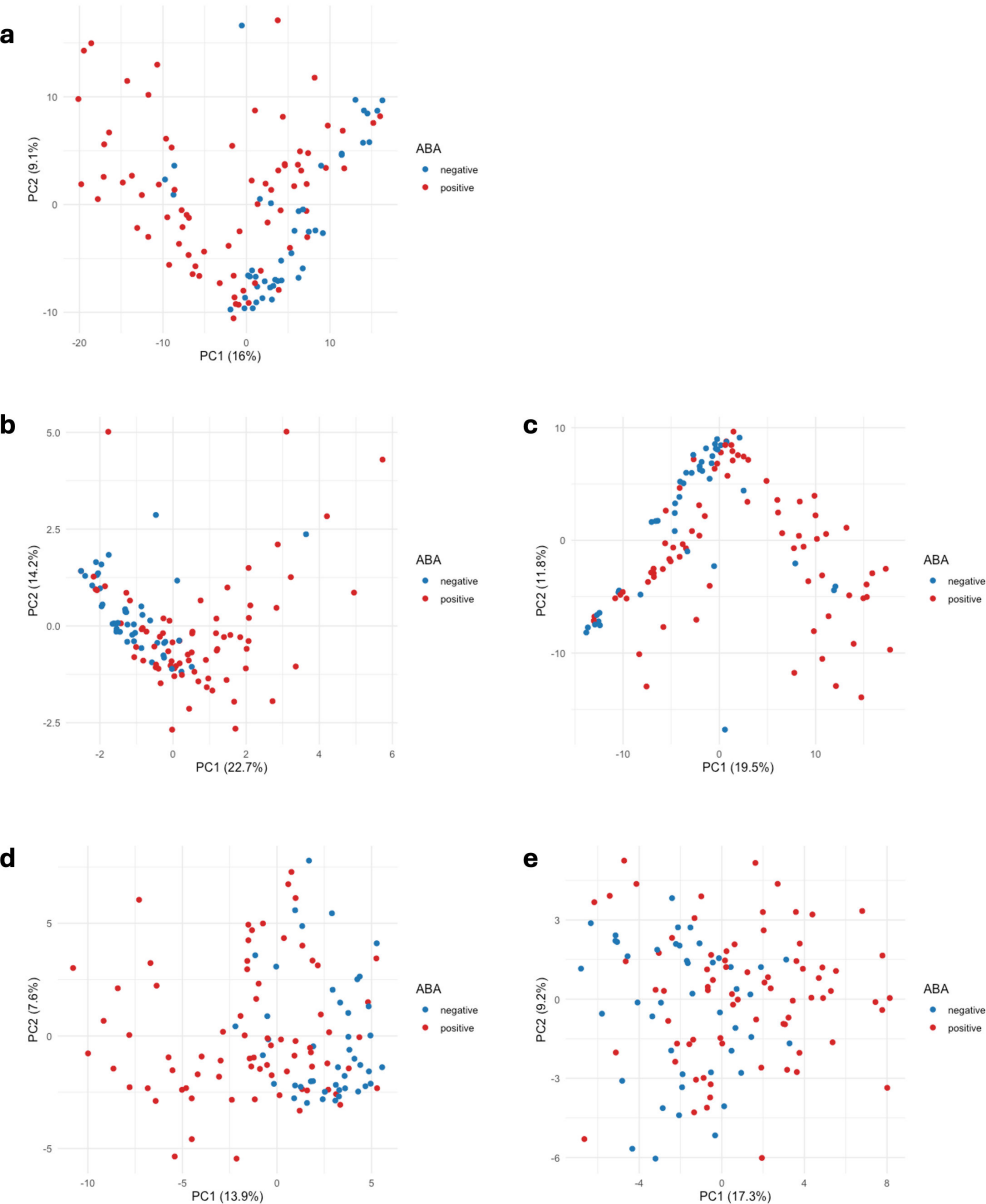
Principal component analysis of the complete or database-based resistome across all databases. To quantitatively assess whether the global resistome structure differed between samples classified as positive or negative by real-time PCR (ABA), multivariate analyses were performed on gene-level abundance matrices derived from the merged resistance table for each database: All combined databases (**a**); ARGA (**b**), CARD (**c**), NCBI database (**d**), and RESF (**e**). PC1 and PC2 highlight separation trends between ABA-positive and ABA-negative samples, indicating that overall resistome composition differs between groups.

To determine whether the global resistome structure differed according to carbapenemase detection (ABA-positive results), multivariate analyses were performed on gene-level abundance matrices derived from the merged resistance table (all databases combined) and from each database separately (CARD, NCBI, RESF, and ARGA). Principal component analysis (PCA) of the complete resistome revealed a clear separation trend between ABA-positive and ABA-negative samples along PC1 (16% of variance) and PC2 (9.1%) (Fig. 4a). Permutational multivariate analysis of variance (PERMANOVA) confirmed a significant difference in overall resistome composition between groups (*P =* 0.001*, R*^2^ *=* 0.042). Inter-group distances were substantially higher than intra-group distances, indicating greater similarity within the same ABA class than across classes. Hierarchical clustering further supported this separation, reproducing ABA classification with high silhouette scores (k2: 0.18) and cluster purity (0.63). This indicates that resistome composition alone is sufficient to discriminate ABA status in an unsupervised framework. Genes contributing most strongly to PCA separation were predominantly associated with multidrug resistance mechanisms, including efflux systems (*Mex*, *Opr*, and *Mux* families), β-lactamases (*bla_OXA_*, *bla_SHV_*, *ADC*, and *ampH*), aminoglycoside-modifying enzymes (*APH* and *AAC*), tetracycline (*tet*), sulfonamide (*sul*), macrolide (*mph*), and trimethoprim (*dfr*) resistance genes. These findings indicate that ABA-positive samples are characterized by a complex multidrug resistome profile rather than by the presence of a single carbapenemase determinant. The same separation pattern was observed when analyses were performed independently for CARD (*P =* 0.001*, R*^2^ *=* 0.040), NCBI (*P =* 0.001*, R*^2^ *=* 0.045), RESF (*P =* 0.001*, R*^2^ *=* 0.037), and ARGA (*P =* 0.001*, R*^2^ *=* 0.070) (Fig. 4b through e), although the magnitude and drivers of separation varied by database. CARD showed the strongest contribution of multidrug resistance determinants, particularly β-lactamases (*bla_OXA_*, *bla_SHV_*, and *bla_KPC_*), aminoglycoside-modifying enzymes, and efflux systems (*Mex*/*Acr* families). In NCBI and RESF, separation was mainly driven by *tet*, *sul*, *mph*, *dfr*, *bla_OXA_*, and efflux-related genes. Even in the sparser ARGA database, clinically relevant genes, such as *APH(3')-VIa*, *bla_SHV-52_*, *bla_OXA-9_*, *ADC*, *tet(A),* and *vanZA,* contributed to sample discrimination.

Although some ABA-negative samples overlapped with ABA-positive samples in the PCA, the subgroup of ABA-positive samples that separated more distinctly along PC1 exhibited a higher overall resistome burden and enrichment of the efflux-associated signature previously described. Differential abundance analysis confirmed that many of these efflux and β-lactamase-associated genes were significantly enriched in ABA-positive samples (*P <* 0.05).

**Fig 5:**
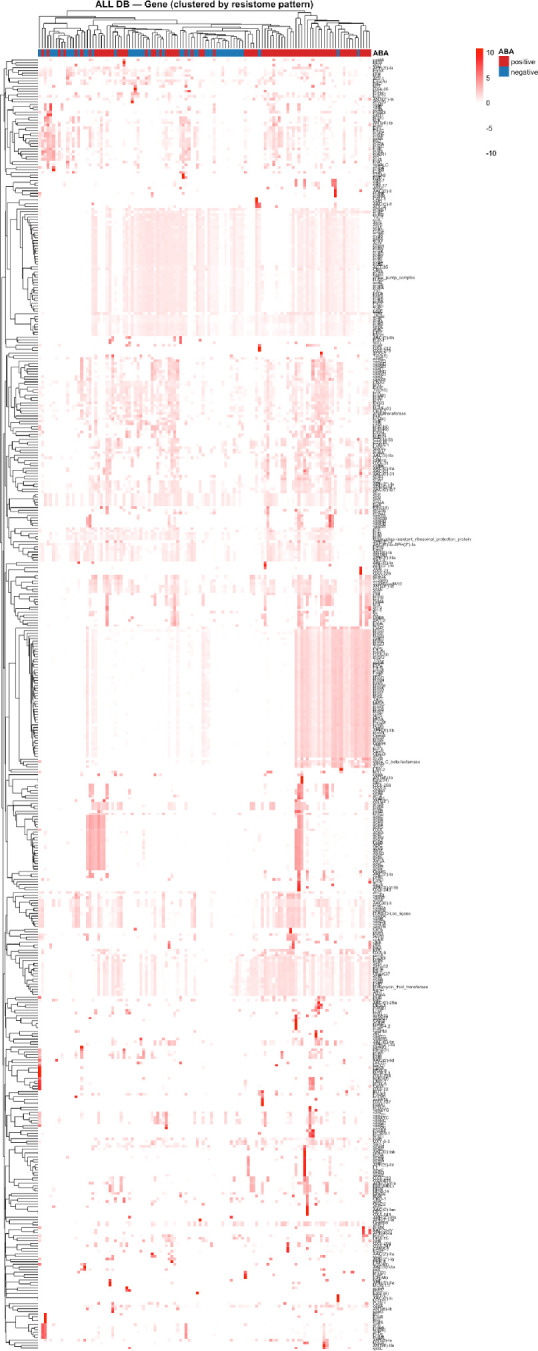
Hierarchical clustering of samples based on resistome gene profiles across all combined reference databases. To investigate whether the overall resistome structure differed between samples classified as positive or negative by real-time PCR (ABA), a resistome pattern analysis was performed using the merged resistance table. For each sample, gene-level counts were transformed into log10(count + 1) values and organized into matrices of gene × sample. Samples were annotated according to their ABA result (positive/negative).

Gene-level hierarchical clustering (Fig. 5) revealed structured organization of samples according to resistome profiles. ABA-positive samples tended to cluster together and were characterized by broader and higher gene abundance patterns, whereas many ABA-negative samples formed distinct clusters with markedly reduced gene representation. Differential abundance analysis identified a large number of genes with significantly different abundances between ABA-positive and ABA-negative samples, supporting the notion that group differences are quantitative and compositional rather than limited to presence/absence of specific carbapenemase genes. Similar patterns were observed in database-specific analyses (Fig. S4 to S7). Within ABA-positive samples, substantial differences in gene detection and abundance were observed across CARD, NCBI, RESF, and ARGA, reflecting database-specific coverage and annotation strategies. In contrast, ABA-negative samples showed fewer inter-database differences, consistent with their overall lower resistome complexity.

## DISCUSSION

AMR represents a major threat to human health, with significant economic and health implications (15). For this reason, several global health organizations have endorsed action plans aimed at ensuring the continued effectiveness of antimicrobial therapies for the treatment of infectious diseases (16). In this context, surveillance plays a pivotal role in informing policies and control strategies. It constitutes the cornerstone for assessing the spread of AMR and for monitoring the impact of local, national, and global interventions (17). Importantly, integrated surveillance systems encompassing humans, animals, plants, and the environment provide a coordinated response within public health and infection control frameworks, while also supporting industrial programs focused on the development of novel antimicrobial agents and diagnostic tools (18).

AMR surveillance is primarily based on the prevalence of ARGs (13). In community-acquired infections, *Enterobacterales* commonly produce extended-spectrum β-lactamases, whereas in hospital settings, metallo-β-lactamases represent a major concern (19). However, active surveillance often relies on bacterial cultures, which require 24–72 h and may underestimate colonization in cases of low bacterial load or fastidious organisms (4), or on targeted molecular assays restricted to predefined genes, potentially missing novel or uncommon variants (20).

While the application of targeted hybrid capture sequencing approach on environmental samples has emerged as a sentinel approach for monitoring the community-level AMR burden (21), investigation of human samples remains limited. In this context, the gastrointestinal tract represents a major reservoir of MDR bacteria, with the intestinal colonization associated with an increased risk of subsequent infection, transmission within healthcare settings, and horizontal mobilization of ARGs within and across species (6).

Within the One Health framework, NGS enables an integrated and untargeted view of the resistome, overcoming the limitations of culture-based and targeted molecular approaches. By providing high-resolution profiling of resistance determinants, NGS has substantially expanded our understanding of resistome complexity, mobility, and ecological distribution. The targeted hybrid capture panel (xHYB) showed high positive agreement when compared to the commercial assay (ABA), particularly for *bla_KPC_* detection, demonstrating strong performance in identifying clinically relevant carbapenemases within complex biological matrices where low target abundance and background microbial diversity limit conventional shotgun sequencing (22, 23). Negative agreement was lower, primarily due to the additional xHYB-positive/ABA-negative detections, especially for *bla_OXA-48_*, which were not deeply characterized. This discrepancy is likely attributable to the identification of multiple *bla_OXA-like_* variants not *bla_OXA-48-like_* that were not correctly targeted by the PCR assay. Notably, DNA concentration did not impair the performance of either ABA or xHYB. Moreover, the inverse correlation between Ct values and sequencing read counts was observed for *bla_KPC_*. To our knowledge, this study represents one of the first attempts to explicitly define and discuss a practical read-count threshold in the context of targeted hybrid capture for AMR surveillance. Hence, we highlighted an important concern regarding the definition of the appropriate threshold for translating metagenomic sequencing signals into clinically interpretable results. The empirical read-count distribution revealed a substantial proportion of low-abundance detections, many of which were not supported by microbiological culture results, suggesting that these signals may reflect background noise or biologically irrelevant detections. However, the use of fixed thresholds in metagenomic antimicrobial resistance analysis remains an open and unresolved methodological issue. Indeed, read counts are influenced by multiple technical and biological factors, including sequencing depth, probe hybridization efficiency, microbial load, and gene copy number. As a result, a universal cutoff may not adequately capture the complexity of resistance gene detection across different targets and sample types. Future studies should aim to refine thresholding strategies through larger data sets, independent validation cohorts, and potentially gene-specific or probabilistic models that better account for the intrinsic variability of metagenomic data.

Interestingly, our results highlight that differences among resistance databases have distinct implications depending on the level of analysis. While aggregation at the gene-family level yields more consistent patterns, gene-level calls may lead to over- or under-interpretation of resistance profiles if not contextualized within broader functional groups. In other words, analyses focusing on precise gene variant identifications (e.g., *bla* subtypes or aminoglycoside-modifying enzyme subclasses) may be significantly impaired. Finally, integrating multiple databases may improve robustness, but careful interpretation remains essential to balance sensitivity and specificity in ARG detection and reporting. Of note, variability in xHYB efficiency across targets may introduce capture bias, potentially affecting the quantitative estimation of gene abundance. While xHYB may improve sensitivity and enable broad and uniform detection across targeted regions, it remains inherently dependent on predefined probe design and reference databases, as we have demonstrated. Detection may be therefore most effective for ARGs and variants with sufficient sequence similarity to the probes, whereas highly divergent sequences may be underrepresented due to reduced capture efficiency and/or limitations in downstream analysis. Importantly, completely novel resistance genes or mechanisms, not previously associated with antimicrobial resistance, need to be identified through untargeted approaches.

Beyond single-gene detection, ABA positivity appears to reflect not merely the presence of a carbapenemase determinant, but a broader reorganization of the resistance gene repertoire, corresponding to a distinct MDR state characterized by coordinated accumulation of diverse resistance mechanisms. Multidrug efflux systems (*Mex, Acr,* and *Mux*), β-lactamases (*bla_OXA_, bla_SHV_,* and *ADC*), aminoglycoside-modifying enzymes (*APH* and *AAC*), and determinants conferring resistance to tetracyclines, sulfonamides, macrolides, and trimethoprim contributed most strongly to group discrimination. The reproducibility of this separation across different databases supports the biological robustness of the signal, indicating that the observed differences reflect a consistent resistome structure rather than database-specific artifacts.

Efflux systems, particularly *Mex*, *Opr*, and *Mux* families, were strongly associated with a resistome differentiation between groups. This aligns with the established role of resistance-nodulation-division (RND) efflux pumps in shaping multidrug resistance phenotypes (24). Clinical isolates of *P. aeruginosa* have been shown to simultaneously overexpress multiple efflux systems, such as *MexAB-OprM* and *MexEF-OprN*, even in the absence of canonical regulatory mutations (24). However, it is important to note that our data do not provide direct evidence of a causal role for these systems in shaping resistome structure. Rather, their consistent enrichment suggests that they may represent markers of broader MDR-associated genomic backgrounds, reflecting coordinated and dynamic accumulation of resistance determinants likely under antibiotic selective pressure. In this context, efflux systems should be interpreted as components of a multidimensional resistance landscape, where their presence is associated with, but not necessarily driving, the observed resistome configuration.

More broadly, the human resistome constitutes a complex and dynamic genetic ecosystem embedded within the microbiome, encompassing genes distributed across both commensal and pathogenic bacteria (25). It represents an ancient and ecologically structured reservoir of ARGs, suggesting that culture-based and PCR-based methods may underestimate emerging resistance determinants. For instance, *Proteobacteria*, particularly *E. coli*, disproportionately contribute to ARG abundance. Although *E. coli* accounts for approximately 40% of ARG abundance in adults, it represents a smaller fraction of the total microbiota (26). Furthermore, resistome composition varies across anatomical niches and is influenced by antibiotic exposure, inflammation, and anthropometric factors (27, 28).

The observation that resistome composition alone is related to ABA “status” suggests that carbapenemase detection may serve as an indirect marker of a broader MDR genomic background. ABA-positive associated strains may represent evolutionarily stabilized MDR lineages in which resistance mechanisms have accumulated in a coordinated manner over time. However, the lack of correlated clinical and ecological data limits our ability to determine the drivers of these resistome shifts and ARG acquisition events.

On the other side, given that plasmid-mediated horizontal gene transfer is a major driver of ARG dissemination (29), understanding the ecological and evolutionary dynamics underlying plasmid emergence in clinical pathobionts remains incomplete (30, 31). Plasmids can move across species and ecological niches under selective pressures, serving as efficient vehicles for ARG mobilization. Consequently, species-specific associations may be misleading and may hinder the development of novel molecular or culture-based detection strategies. Notably, disease-associated microbiomes often display expanded resistomes despite modest taxonomic shifts, indicating that resistance changes may occur at the strain level or through acquisition of mobile ARGs without major compositional alterations (32).

Collectively, growing evidence on the microbial ecosystems supports a broader “pan-resistome” concept: within this framework, an ARG-centric rather than species-centric approach becomes more appropriate. Surveillance strategies are progressively shifting toward detection of resistance genes and mutations themselves, reflecting the biological reality that ARGs are mobile genetic elements capable of horizontal transfer across species and ecological niches. Therefore, surveillance should prioritize tracking highly mobile and clinically relevant ARGs and their vectors rather than focusing exclusively on pathogenic species.

In this context, antimicrobial susceptibility testing is progressively moving toward molecular susceptibility tests (13). By leveraging NGS-based or analogous molecular technologies, resistance profiling can be achieved directly through detection of ARGs, significantly reducing turnaround times compared with phenotypic methods. This shift is particularly relevant not only to optimize antimicrobial therapy, but also in prevention strategies.

Finally, our findings highlight the intrinsic challenges of translating metagenomic sequencing signals into clinically interpretable diagnostic results for antimicrobial resistance detection. Although NGS enables the comprehensive detection of resistance determinants, the quantitative nature of sequencing data introduces important analytical considerations when defining positivity thresholds. The use of a fixed read-count cutoff resulted in a good concordance with the reference molecular assay. However, a threshold-based classification strategy may require optimization or gene-specific calibration in metagenomic diagnostic workflows. Future work should therefore focus on integrating additional parameters to improve the interpretability and diagnostic performance of metagenomic AMR detection.

## MATERIALS AND METHODS

### Sample manipulation and culture method

An aliquot of each rectal swab specimen was resuspended in sterile phosphate-buffered saline (PBS) and plated on Tryptic Soy Agar (TSA; VWR, Leuven, Belgium), MacConkey agar (MAC; Oxoid, Basingstoke, UK), and chromID CARBA SMART Agar (bioMérieux, France) containing a mixture of antibiotics which enables the selective growth of *Klebsiella pneumoniae carbapenemase* (*KPC*) and metallo-carbapenemase-producing bacteria for the CARB medium and OXA-48-type-producing bacteria for the OXA medium. The seeded plates were incubated at 37°C ± 0.5°C for 18–24 h; colonies were then identified using Matrix-Assisted Laser Desorption/Ionization Time-of-Flight (MALDI-TOF, Bruker Daltonics, Bremen, Germany) as described above (13). DNA was isolated from 120 fecal specimens (Data S1) using the DANAGENE MICROBIOME Swab DNA Kit (Danagene, Spain), following the manufacturer’s protocol. Extracted DNA was stored at −20°C and quantified before sequencing using Nanodrop One (ThermoFisher).

### Detection of ARGs by AB Analitica real-time PCR

Rectal swabs were vortexed and an aliquot was processed using the REALQUALITY Carba-Screen assay (AB Analitica, Italy), a molecular diagnostic kit designed for the detection of carbapenem resistance genes at two levels of identification: the assay can be used both for patient screening and as a gene confirmatory molecular test. The workflow includes an initial screening step (Mix Carba-Screen) to identify samples positive for class A, B, and D carbapenemases, as well as *AcOXA* genes. Samples testing positive at this stage undergo a second-step analysis for specific gene identification using Mix Carba B (*bla_VIM_*, *bla_NDM_*, and *bla_IMP_*) and Mix Carba A + D (*bla_KPC_* and *bla_OXA-48_*).

### Detection of ARGs by QIAseq xHYB AMR panel

Sequencing libraries were generated from extracted DNA using the QIAseq FX DNA Library Kit (Qiagen, Hilden, Germany) where unique dual index adapters were included and used for multiplexing following the manufacturer’s instruction. Libraries were pooled and enriched for ARGs by multiplex hybrid capture using the QIAseq xHYB AMR Panel Kit (Qiagen, Hilden, Germany). Briefly, purified libraries were pooled, and an enhanced blocking buffer added to prevent non-specific hybridization. Libraries were concentrated using QIAseq beads, mixed with the QIAseq xHYB probe panel, and incubated at 70°C for 16 h. Hybridized libraries were captured on streptavidin beads and washed twice to remove unbound DNA. After elution, the enriched libraries were PCR amplified for 20 cycles. The final library pool was sequenced on an AVITI LT Instrument (Element Biosciences, California, San Diego, USA) using the AVITI Cloudbreak Sequencing Kits in the 2 × 150 bp PE mode (Element Biosciences, California, San Diego, USA).

Data were basecalled, manually demultiplexed, and converted to FASTQ format as suggested by the manufacturer. Data were then analyzed using the “Find QIAseq xHYB AMR Markers” workflow in CLC Genomics Workbench v. 24.0 (Qiagen, Hilden, Germany) using default parameters. The Qiagen QMI-AR Peptide Marker Database (v. 2021-08) (qmi_ar_peptide_ marker_database_2021_08), which covers 3.622 antimicrobial resistance genes, was used as recommended. The database contains peptide markers derived from the following sources: The CARD, NCBI database, RESF, and ARG ANNOT (ARGA). Briefly, raw reads were trimmed, removing adapter sequences and low-quality nucleotide sequences. Resistance genes were then detected and quantified using the ShortBRED algorithm (33) with the genetic code set to 11 (Bacterial, Archaeal, and Plant Plastid), *E*-value at 0.00,001, 95% identity, minimum alignment length 0.95, minimum read length 90.0, and running in the more sensitive search mode. Data were reported in number of reads as well as normalized abundance in RPKM (Reads Per Kilobase per Million reads).

### Statistical analysis

All analyses were performed using R Statistical Software v4.1.2 (34). A merged resistance annotation table was generated in which each record corresponded to a peptide marker and included the following fields: *Peptide_Marker_ID*, *Gene*, *Gene_Family*, *Peptide_Marker_DB,* and sample-specific abundance columns reporting either raw read counts or normalized expression values (RPKM) (Data S1). For each database (ARGA, CARD, NCBI, and RESF), the number of distinct peptide markers, genes, gene families, Gene ARO annotations, and Phenotype ARO annotations was determined using unique counts. Differences in the distribution of peptide-marker support across databases were assessed using chi-square tests, with effect sizes quantified by Cramér’s *V*. For each peptide marker, total abundance was calculated by summing values across all sample-specific columns, separately for read counts and RPKM. These values were subsequently aggregated at the gene and gene family levels by summing the abundances of all peptide markers mapping to the same entity within each database. Associations between peptide-marker support and abundance were evaluated at both the gene and gene family levels, and for both abundance metrics (counts and RPKM). For each database, the number of peptide markers identifying a given gene or gene family was correlated with the corresponding total abundance using Spearman’s rank correlation. Correlation coefficients (ρ), *P-*values, and BH-adjusted *P-*values were reported.

To convert sequencing read counts into binary detection calls, a positivity threshold of ≥10 reads per gene family was applied. This threshold was empirically defined based on the observed read count distribution, which showed a high proportion of low-abundance detections clustered in the single-digit range (1–10 reads), likely reflecting background sequencing noise. Furthermore, low read-count detections were frequently associated with negative microbiological culture results, supporting their interpretation as low-confidence signals rather than clinically meaningful ARG detection. The ≥10 read threshold was therefore adopted as a conservative cutoff to enhance interpretability.

A gene-level concordance analysis was performed for the main carbapenemase targets (*bla_KPC_, bla_OXA-48_, bla_VIM_,* and *bla_NDM_*) by comparing metagenomic detection (xHYB) with real-time PCR results (ABA), which were used as the reference standard. For each gene, a 2 × 2 confusion matrix was constructed, and agreement metrics were calculated, including overall agreement, Cohen’s kappa coefficient, PPA, NPA, PPV, and NPV. For *bla_KPC_, bla_OXA-48_, bla_NDM_*, the distribution of log-transformed read counts was compared between ABA-positive and ABA-negative samples. Given the non-normal and zero-inflated nature of the data, group differences were assessed using the Wilcoxon rank-sum test. Resulting *P-*values were adjusted for multiple testing as described above. To evaluate whether sequencing depth of target genes was influenced by DNA input quantity, correlations between log-transformed read counts and DNA concentration were assessed using Spearman’s rank correlation test. *P-*values were adjusted using the BH method.

To investigate differences in overall resistome structure between ABA-positive and ABA-negative samples, a resistome pattern analysis was conducted. Gene-level counts were log transformed and organized into gene-by-sample and gene-family-by-sample matrices. Within each database (combined databases, CARD, NCBI, RESF, and ARGA), comparisons between ABA-positive and ABA-negative samples were performed at both gene and gene family levels using the Wilcoxon rank-sum test for abundance data. In addition, Fisher’s exact test was applied to presence/absence data to assess associations between gene detection and ABA classification.

Between-database comparisons were conducted separately for ABA-positive and ABA-negative samples. Gene abundance differences across databases were evaluated using the Kruskal-Wallis test, while differences in detection frequencies were assessed using chi-square or Fisher’s exact tests, as appropriate.

To quantitatively assess differences in global resistome structure between ABA-positive and ABA-negative samples, multivariate analyses were performed on gene-level abundance matrices. For each database, gene counts were log-transformed and row-scaled to emphasize relative resistome profiles. Euclidean distance matrices were computed, and PERMANOVA was used to test for significant differences in resistome composition between groups. Hierarchical clustering was performed, and silhouette scores were calculated to evaluate how well unsupervised clustering recapitulated ABA classification. Cluster purity was also assessed to quantify concordance between clustering results and ABA labels. Mean intra- and inter-group distances were calculated to further characterize resistome similarity within and between groups. Finally, PCA was used to visualize resistome structure.

Two complementary statistical approaches were applied: within-database comparison (ABA positive vs. negative) and between-database comparison (each database versus each other).

Heatmaps display log10-transformed gene counts scaled by row to emphasize resistome patterns rather than absolute abundance. Columns represent individual samples, annotated by ABA real-time PCR result (positive/negative). Rows represent resistance genes detected in each database. Hierarchical clustering was applied to both samples and genes. Across all databases, samples tend to cluster according to ABA result, indicating that the global resistome structure is strongly associated with molecular detection of carbapenemase genes. The separation is most evident in CARD, followed by NCBI and RESF, and remains detectable in ARGA despite lower gene density (see Fig. S2 to S5).

## Data Availability

The sequence data generated in this study have been deposited in the European Nucleotide Archive (ENA) under the project accession number PRJEB113311. The original contributions presented in the study are included in the article; further inquiries can be directed to the corresponding author.

## SUPPLEMENTAL MATERIAL

Data S1 msphere.00221-26-s0001.xlsx Antimicrobial resistance genes detected by using hybrid capture across samples.

Data S2 msphere.00221-26-s0002.csv Antimicrobial resistance genes for each database.

Data S3 msphere.00221-26-s0003.csv Antimicrobial resistance gene families for each database.

Supplemental Figures msphere.00221-26-s0004.pdf Figures S1 to S7.

Supplemental Legends msphere.00221-26-s0005.docx Supplemental data and figure legends.

ASM does not own the copyrights to Supplemental Material that may be linked to, or accessed through, an article. The authors have granted ASM a non-exclusive, world-wide license to publish the Supplemental Material files. Please contact the corresponding author directly for reuse.
