# Supplementary material for: From gene detection to resistome ecology: targeted hybrid capture in AMR surveillance
Source: mSphere. 2026 Jun 15;11(7):e00221-26. doi: 10.1128/msphere.00221-26 (PMC13410987; doi:10.1128/msphere.00221-26)

# Supplementary Figure 1

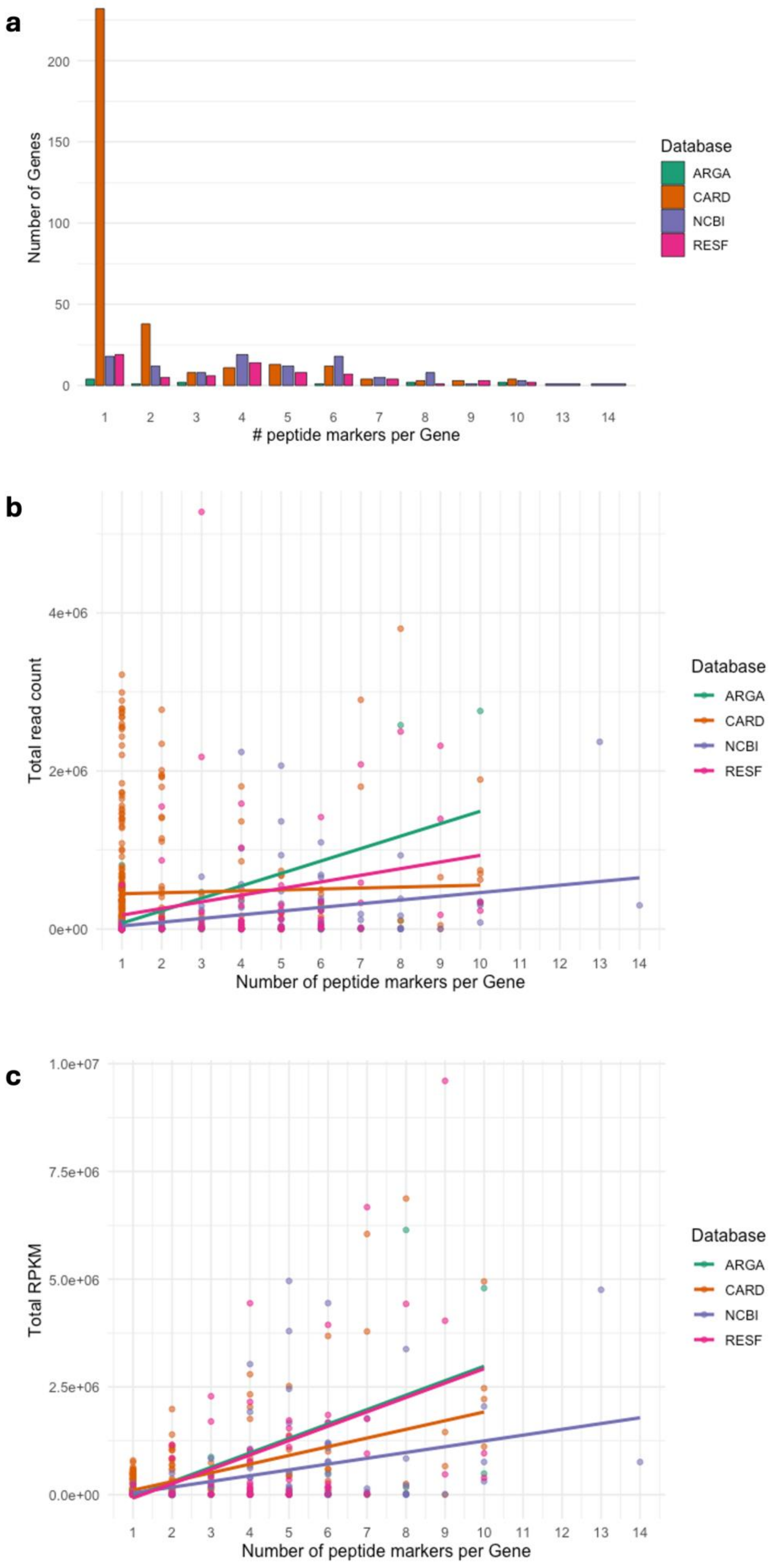

# Supplementary Figure 2

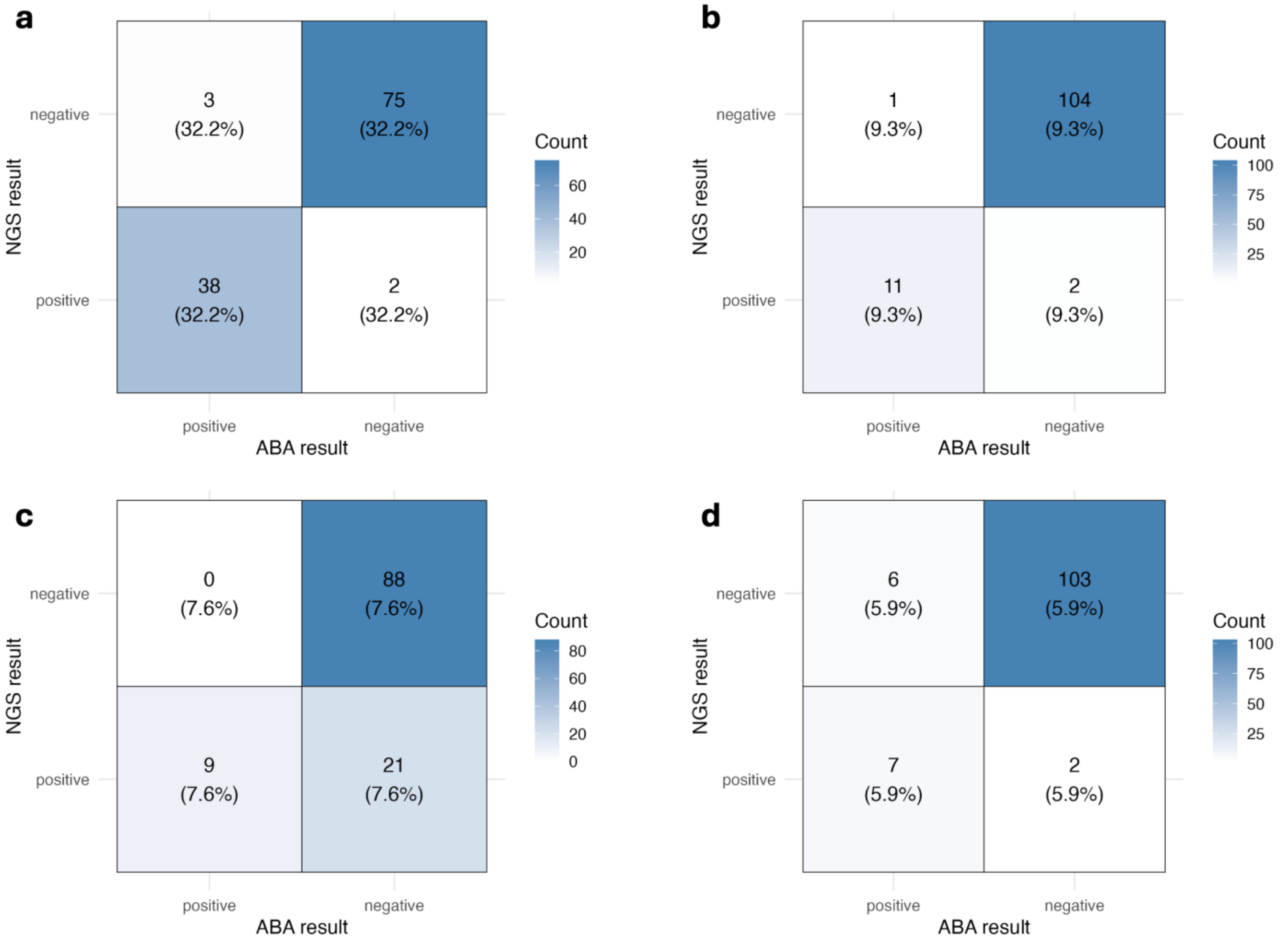

# Supplementary Figure 3

a

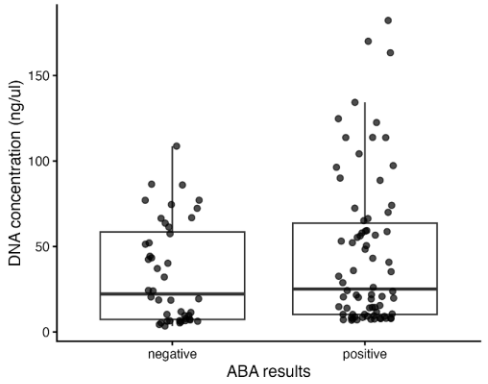


b

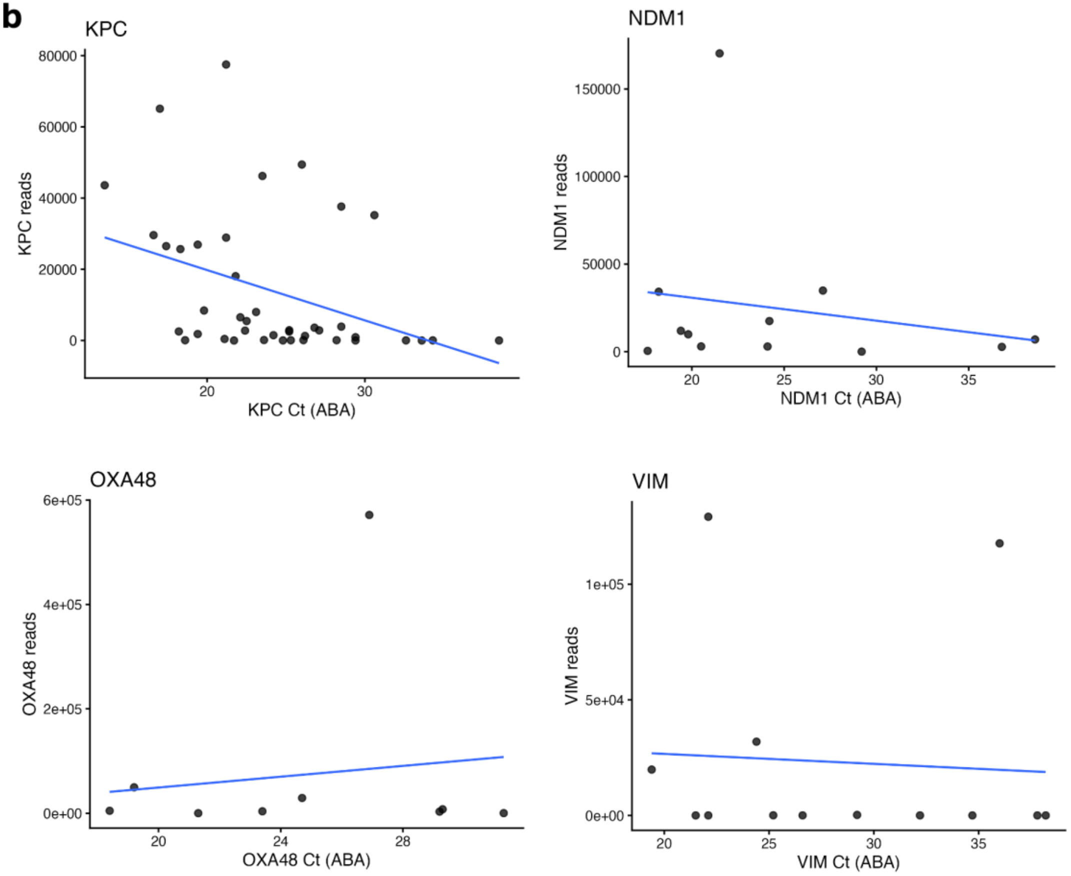

# Supplementary Figure 4

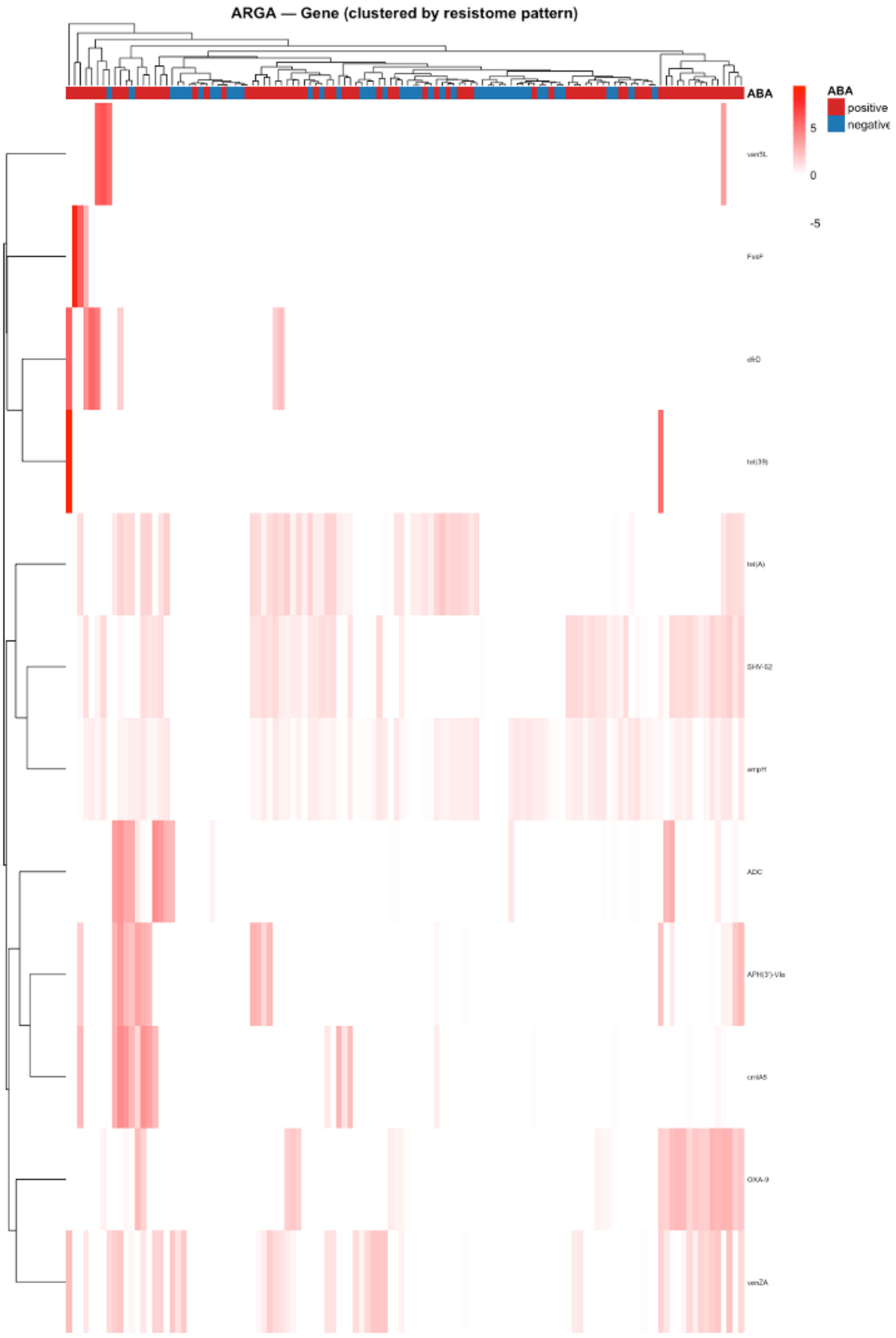

# Supplementary Figure 5

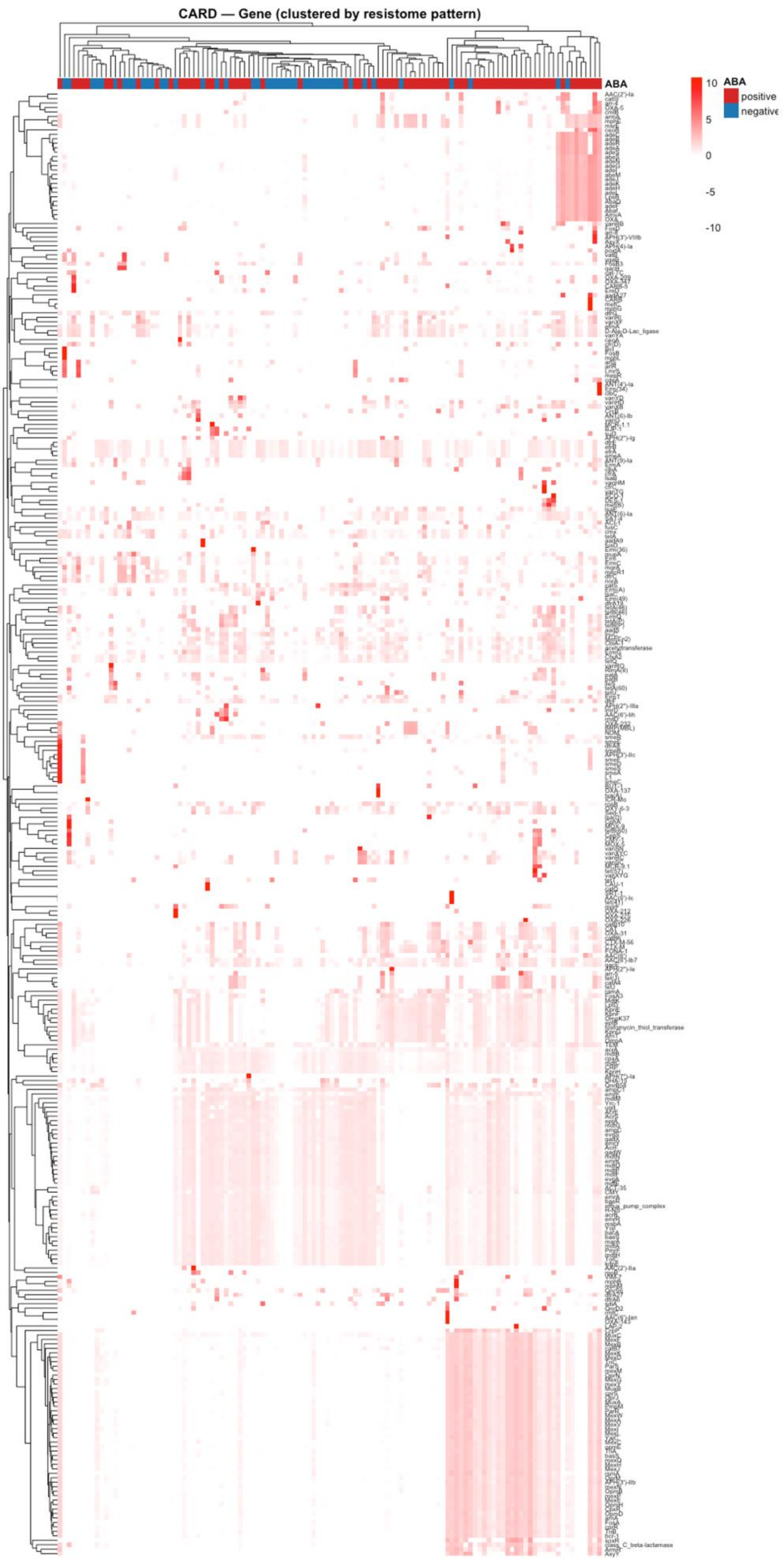

# Supplementary Figure 6

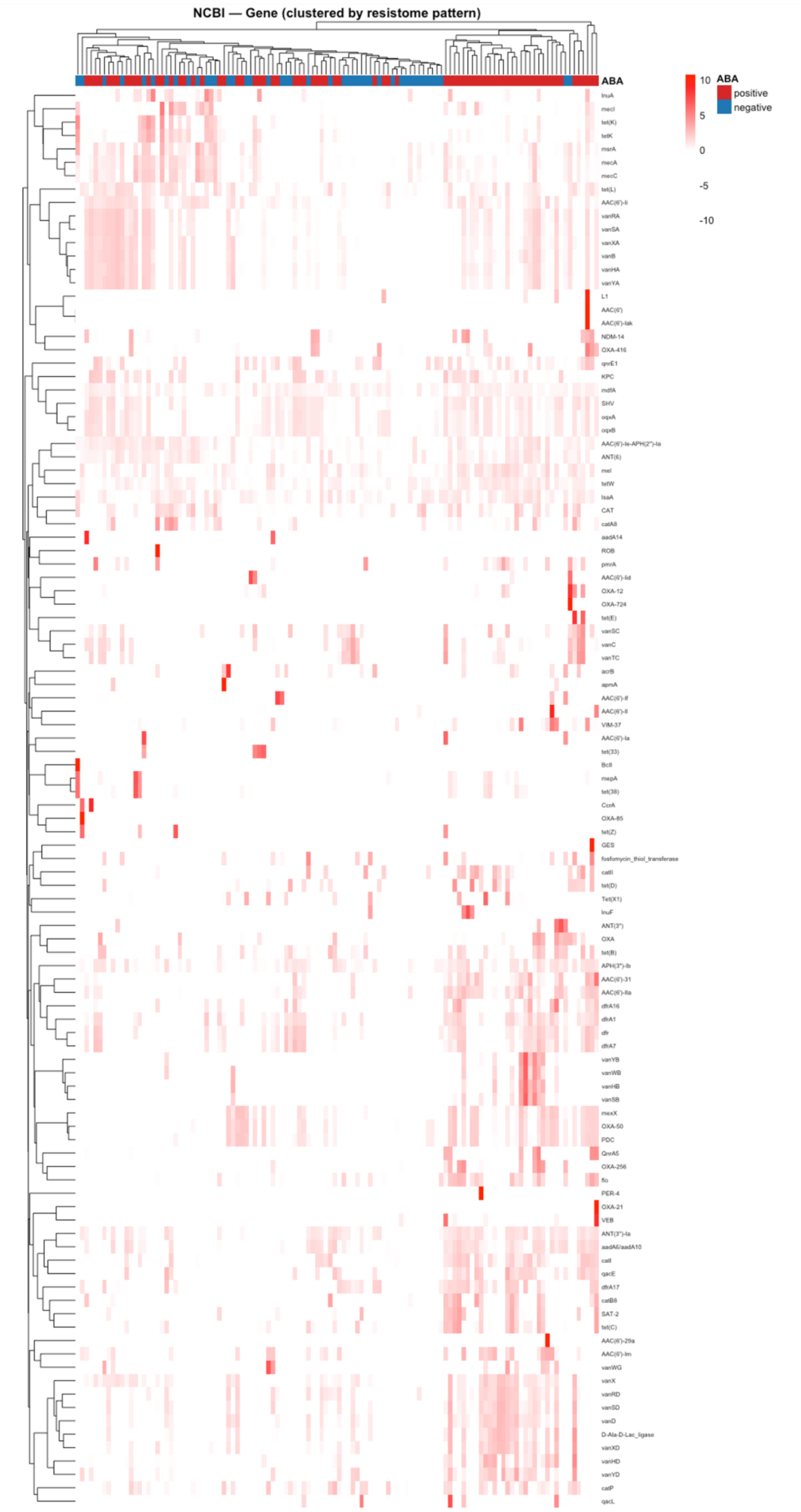

# Supplementary Figure 7

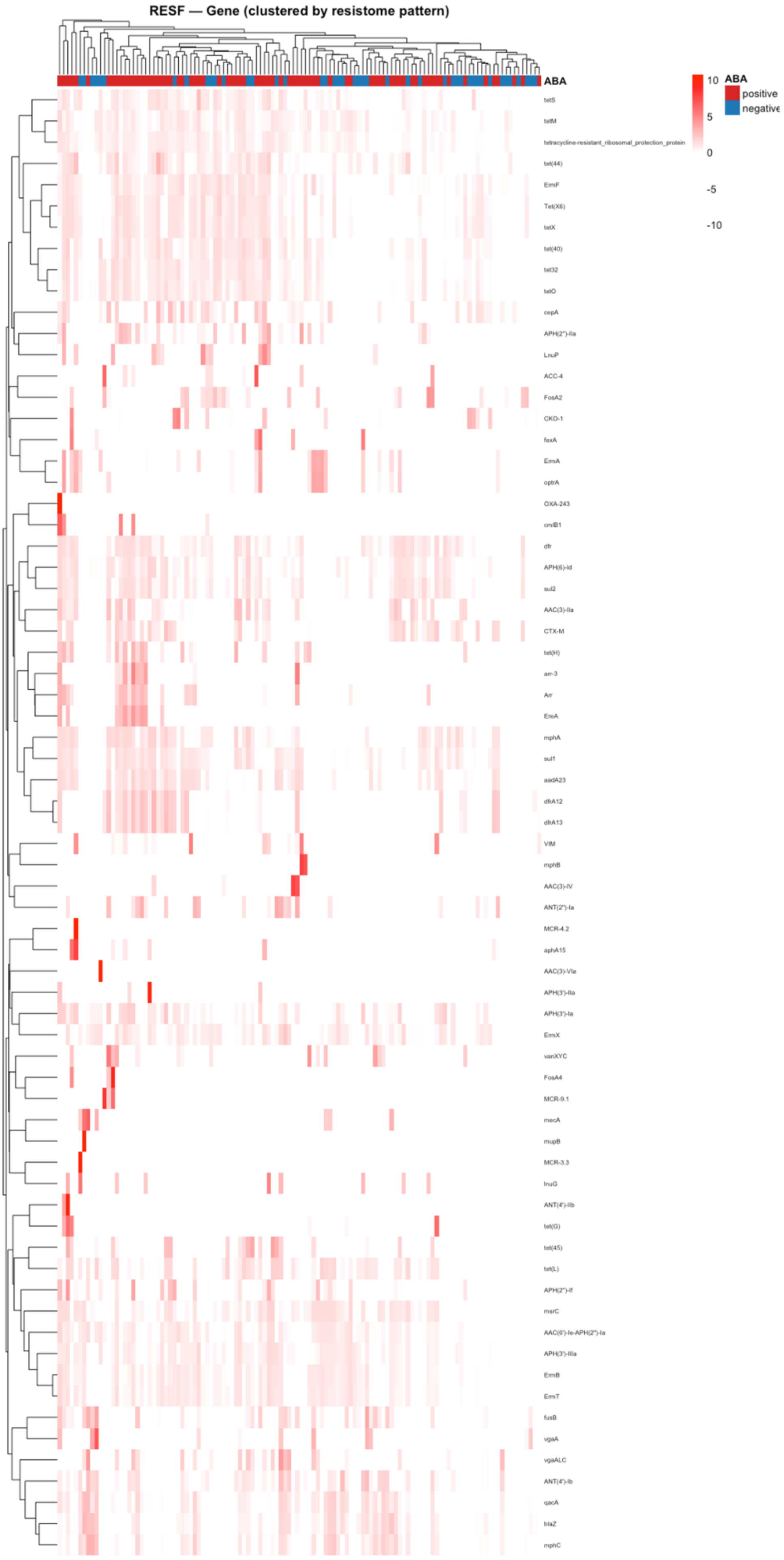

Supplement: Supplemental Figures — Figures S1 to S7. [file msphere.00221-26-s0004.pdf]
