# Supplementary material for: From gene detection to resistome ecology: targeted hybrid capture in AMR surveillance
Source: mSphere. 2026 Jun 15;11(7):e00221-26. doi: 10.1128/msphere.00221-26 (PMC13410987; doi:10.1128/msphere.00221-26)
Supplement: Supplemental Legends — Supplemental data and figure legends. [file msphere.00221-26-s0005.docx]

**Supplementary Materials**

**Supplementary Data 1.** List of antimicrobial resistance genes detected by using hybrid capture (xHYB) across samples. The table reports peptide marker identifiers, associated genes and gene families, together with raw counts and normalized abundance values (RPKM) for each sample as obtained following CLC Genomics analysis.

**Supplementary Data 2.** List of antimicrobial resistance genes for each database (ARG analyzer, ARGA; The Comprehensive Antibiotic Resistance Database, CARD; National Center for Biotechnology Information database, NCBI and ResFinder database, RESF) and shared between them.

**Supplementary Data 3.** List of antimicrobial resistance gene families for each database (ARG analyzer, ARGA; The Comprehensive Antibiotic Resistance Database, CARD; National Center for Biotechnology Information database, NCBI and ResFinder database, RESF) and shared between them. Gene-level and gene-family summaries for clinically relevant resistance markers detected across samples. The table reports the number of samples in which each gene or gene family was detected, together with count statistics and database annotations.

**Supplementary Figure 1. (a) Distribution of peptide-marker support for ARGs.** To assess peptide-marker support, the number of distinct Peptide Markers was associated with each ARG and computed for each database (ARG analyzer, ARGA; The Comprehensive Antibiotic Resistance Database, CARD; National Center for Biotechnology Information database, NCBI and ResFinder database, RESF). Distributions were summarized as the number of genes identified by 1 or more peptide markers. These distributions were visualized using grouped bar plots, with bars coloured by database. Differences in the distributions of peptide-marker support across databases were evaluated using chi-square tests of independence. Effect sizes were quantified using Cramér’s V. **(b) Gene level correlation between peptide-marker support and total read counts.** Scatter plot showing the relationship between the number of peptide markers identifying a gene and the total read counts. Database-specific trend lines highlight differences in correlation strength. **(c) Gene level correlation between peptide-marker support and total RPKM.** Scatter plot showing the relationship between peptide-marker support and normalized abundance (RPKM) at the gene level across databases. For each peptide marker, total abundance was calculated by summing values across all sample-specific columns, separately for read counts and for RPKM. These totals were aggregated at the gene level by summing abundances across all peptide markers mapping to the same entity within each database. Associations between peptide-marker support and abundance were evaluated at gene level and for both read counts and total RPKM by using Spearman rank correlation.

**Supplementary Figure 2. Confusion matrix comparing hybrid capture (xHYB) detection of carbapenemase genes (xHYB) showing more than 10 reads with the commercial AB analitica (ABA) real-time.**

xHYB detection of carbapenemase genes was compared against the validated real-time PCR AB Analitica (ABA) assay (AB) targeting the same resistance determinants (i.e. *bla_KPC_, bla_OXA-48-like_, bla_VIM_, bla_NDM_, blaI_MP_*). ARGs detected by NGS were summarized into a binary variable and defined as positive when at least one target gene showed read counts > 10 and negative otherwise. ABA results were recorded as positive, negative, or not determined (ND). Samples with ND results in either method were excluded from the concordance analysis. Concordance analysis was performed for each carbapenemase gene *bla_KPC_* **(a)**, *bla_NDM_* **(b)** and *bla_OXA-48-like_* **(c)**, *bla_VIM_* **(d)**, The heatmap shows the distribution of samples classified as positive or negative by both methods. ABA was considered the reference test. Numbers inside each cell represent the absolute count of samples, with percentages calculated over the total number of evaluable samples. True positives and true negatives accounted for an overall agreement of 77.97%, with a Cohen’s kappa of 0.506, indicating moderate concordance between the two molecular approaches.

**Supplementary Figure 3. DNA concentration according to ABA test results. (a)** Boxplots with overlaid jittered points showing the distribution of the extracted DNA concentration (ng/µL) in samples classified as ABA negative or ABA positive. Each point represents an individual sample, the central line indicates the median, and the box represents the interquartile range. **(b) NGS read counts according to ABA test results.** Scatter plots illustrating the relationship between ABA PCR Ct values and NGS read counts for *bla_KPC_*, *bla_NDM1_*, *bla_OXA-48_*, and *bla_VIM_*. A regression line is included to visualize the overall trend. Spearman correlation analysis revealed a significant inverse association for *bla_KPC_* (ρ = 0.468, p = 0.002), while correlations for *bla_NDM_* (ρ = 0.154, p = 0.633), *bla_OXA-48-like_* genes (ρ = 0.150, p = 0.700), and *bla_VIM_* (ρ = 0.314, p = 0.295) were not statistically significant.

**Supplementary Figure 4. Hierarchical clustering of samples based on resistome gene profiles obtained using The Comprehensive Antibiotic Resistance Database.** Heatmap display log10-transformed gene counts scaled by row to emphasize resistome patterns rather than absolute abundance. Columns represent individual samples, annotated by ABA real-time PCR result (positive/negative). Rows represent resistance genes detected in CARD database. Hierarchical clustering was applied to both samples and genes.

**Supplementary Figure 5. Hierarchical clustering of samples based on resistome gene profiles obtained using National Center for Biotechnology Information database.** Heatmap display log10-transformed gene counts scaled by row to emphasize resistome patterns rather than absolute abundance. Columns represent individual samples, annotated by ABA real-time PCR result (positive/negative). Rows represent resistance genes detected in NCBI database. Hierarchical clustering was applied to both samples and genes.

**Supplementary Figure 6. Hierarchical clustering of samples based on resistome gene profiles obtained using ARG analyzer.** Heatmap display log10-transformed gene counts scaled by row to emphasize resistome patterns rather than absolute abundance. Columns represent individual samples, annotated by ABA real-time PCR result (positive/negative). Rows represent resistance genes detected in ARGA database. Hierarchical clustering was applied to both samples and genes.

**Supplementary Figure 7. Hierarchical clustering of samples based on resistome gene profiles obtained using ResFinder database.** Heatmap display log10-transformed gene counts scaled by row to emphasize resistome patterns rather than absolute abundance. Columns represent individual samples, annotated by ABA real-time PCR result (positive/negative). Rows represent resistance genes detected in RESF database. Hierarchical clustering was applied to both samples and genes.
